# Characterisation of three novel α-L-arabinofuranosidases from a compost metagenome

**DOI:** 10.1186/s12896-019-0510-1

**Published:** 2019-04-18

**Authors:** Brent Fortune, Sizwe Mhlongo, Leonardo Joaquim van Zyl, Robert Huddy, Mariette Smart, Marla Trindade

**Affiliations:** 1 Institute for Microbial Biotechnology and Metagenomics, University of the Western Cape, Bellville, South Africa; 2 Centre for Bioprocess Engineering Research, University of Cape Town, Cape Town, Western Cape South Africa

**Keywords:** Thermostability, Arabinofuranosidase, Compost, Metagenomics

## Abstract

**Background:**

The importance of the accessory enzymes such as α-L-arabinofuranosidases (AFases) in synergistic interactions within cellulolytic mixtures has introduced a paradigm shift in the search for hydrolytic enzymes. The aim of this study was to characterize novel AFase genes encoding enzymes with differing temperature optima and thermostabilities for use in hydrolytic cocktails.

**Results:**

Three fosmids, pFos-H4, E3 and D3 were selected from the cloned metagenome of high temperature compost, expressed in *Escherichia coli* and subsequently purified to homogeneity from cell lysate. All the AFases were clustered within the GH51 AFase family and shared a homo-hexameric structure. Both AFase-E3 and H4 showed optimal activity at 60 °C while AFase-D3 had unique properties as it showed optimal activity at 25 °C as well as the ability to maintain substantial activity at temperatures as high as 90 °C. However, AFase-E3 was the most thermostable amongst the three AFases showing full activity even at 70 °C. The maximum activity was observed at a pH profile between pH 4.0–6.0 for all three AFases with optimal activity for AFase H4, D3 and E3 at pH 5.0, 4.5 and 4.0, respectively. All the AFases showed K_*M*_ range between 0.31 mM and 0.43 mM, K_cat_ range between 131 s^− 1^ and 219 s^− 1^ and the specific activity for AFase-H4, AFases-E3 and was 143, 228 and 175 U/mg, respectively. AFases-E3 and D3 displayed activities against pNP-β-L-arabinopyranoside and pNP-β-L-mannopyranoside respectively, and both hydrolysed pNP-β-D-glucopyranoside.

**Conclusion:**

All three AFases displayed different biochemical characteristics despite all showing conserved overall structural similarity with typical domains of AFases belonging to GH51 family. The hydrolysis of cellobiose by a GH51 family AFase is demonstrated for the first time in this study.

**Electronic supplementary material:**

The online version of this article (10.1186/s12896-019-0510-1) contains supplementary material, which is available to authorized users.

## Background

Hemicellulose is a highly branched hetero polymer which represents approximately one third of the total dry weight of plant biomass [1]. Heteroxylan forms the backbone of hemicellulose with xylopyranosyl residues linked by β-1, 4-glycosidic bonds which are mainly substituted with arabinose or glucuronic acid at their 2-O and/or 3-O positions [2, 3]. L-arabinosyl residues are mainly associated with arabinan, arabinoxylan, gum Arabic and arabinogalactan [4]. These residues participate in the crosslinking within the plant cell wall structure and thus strongly inhibit the degradation of xylan to simple xylose sugar units by xylan-degrading enzymes [5]. It is estimated that 40 million tons of xylan-rich agro-industrial biomass is generated globally on an annual basis. The potential of xylan-rich biomass feedstock has triggered an increasing interest in bioprospecting efforts for enzymes to degrade them to monomeric sugars which can be converted or used as part of raw materials in the production of value added products such as bioplastics, biodiesel and bioethanol [6].

The composition of enzyme cocktails largely depends on the type of biomass to be hydrolysed, the complexity of the bonds and carbohydrate structures [3]. Enzyme cocktails for biomass degradation typically include endoglucanases (EC 3.2.1.4), cellobiohydrolases (EC 3.2.1.91), β-glucosidases (EC 3.2.1.21) and a variety of hemicellulases. The core hemicellulases include endo-β-xylanases (EC 3.2.1.8), β-1,4-D-xylosidases (EC 3.2.1.37) and α-L-arabinofuranosidases (EC 3.2.1.55). These hemicellulases synergistically hydrolyse hemicellulose where α-L-arabinofuranosidase is involved in the hydrolysis of α-1,2 and α-1,3 glycosidic bonds that link α-arabinofuranoyl side moieties while endo-β-xylanases and β-1,4-D-xylosidases act on the β-1,4-bonds that link D-xylosyl residues [7].

Extreme environments such as hot springs, deep sea hydrothermal vents and organic composts are reservoirs of unique microbial diversity, providing the potential for isolating novel enzymes with desirable industrial properties [8]. The adaptation of microbial communities to these environmental conditions explains their high genomic and metabolic flexibility, and they often encode enzymes with properties suitable for many industrial applications [9]. Frequently, enzymes from these environments have been shown to be robust catalysts able to withstand high temperatures which are used to promote opening of the structure of lignocellulosic material. This not only leads to better enzyme penetration and therefore cell wall degradation but reduces or eliminates the need for cooling of the material prior to enzymatic pre-treatment [8]. This has the potential of increasing reaction rates by several orders of magnitude resulting in reduced enzyme loading and time required for efficient hydrolysis and saccharification, thus reducing the cost of the overall production process [8, 10, 11].

Using function-based metagenomic screening approaches, novel sequences from both cultivable and uncultivable microorganisms can be exploited without prior sequence information [6]. This screening approach thus provides access to previously unknown genes and their novel enzymes with unique structural and kinetic properties [12, 13]. Ohlhoff et al. [14] constructed a fosmid metagenomic library from high temperature compost which was subsequently screened for several classes of lignocellulases [15]. In this study, three AFases were chosen from these initial screens and were characterized with the aim of bioprospecting for unique biochemical properties that could be of use in industrial applications.

## Methods

### Bacterial strains and plasmids used in this study

Bacterial strains and plasmids used in this study are listed in Table 1 and primers are listed in Table 2. Restriction endonucleases and T4 DNA ligase were purchased from Fermentas (ThermoFisher Scientific) and used following the manufacturer’s instructions. Unless otherwise stated, all *E. coli* strains were maintained in Luria broth (LB) or agar (LA) (Sigma Aldrich, South Africa) and cultivated at 37 °C with shaking at 160 rpm. *E. coli* strains transformed with pJET1.2 and pET21a plasmids were cultured in medium supplemented with 100 μg/mL ampicillin. *E. coli* strains transformed with pCC1Fos™ were cultured in medium supplemented with 15 μg/mL chloramphenicol while a double selection with 15 μg/mL chloramphenicol and 50 μg/mL kanamycin was applied for the transposon treated fosmid constructs.

**Table 1:** Bacterial strains, plasmid vectors and their recombinant versions used in this study

Strain	Genotype^a^	Reference
*E. coli* Epi300	F-mcrA D(mrr-hsdRMS-mcrBC) f80dlacZDM15 DlacX74recA1 endA1 araD139 (ara, leu) 7697 galK1-rspLnupGtrfr	Invitrogen, USA
Genehog	F-mcrA ∆(mrr-hsdRMS-mcrBC) _φ_80dlacZ∆M15 ∆lacX74recA1 araD139 ∆(ara, leu) 7697 galUgalKrpsL (StrR(endA1 nupGfhuAIS2r	Epicentre Biotechnology, USA
BL21 (DE3)	F-ompThsdSB(rB-mB-)gal dcm gal ʎ(DE3)	Invitrogen, USA
pCC1Fos™	pCC1Fos™L-Arabinose inducible promoter Copy Control; Cam^R^, F factor ori, oriV high copy ori, λcos site for λ packaging, Bacteriophage T7 RNA polymerase promoter	Invitrogen, USA
pFos-H4	pCC1FOS containing 17.5 kb of cloned metagenomic DNA as an insert with AFase activity, Cam^R^	IMBM
pFos-E3	pCC1FOS containing 20.7 kb of cloned metagenomic DNA as an insert with AFase activity, Cam^R^	Dr C. Ohlhoff, IMBM, UWC, SA
pFos-D3	pCC1FOS containing 10. 7 kb of cloned metagenomic DNA as an insert with AFase activity, Cam^R^	Dr C. Ohlhoff, IMBM, UWC, SA
pJET 1.2/blunt	Suicide cloning vector (eco47IR), blunt DNA ends for ligation with insert, T7 promoter, Amp^R^	Fermentas, USA
pJET-H4	1467 bp AFase-H4 gene amplicon blunt-end ligated into pJet1.2	This study
pJET-E3	1547 bp AFase-E3 gene amplicon blunt-end ligated into pJet1.2	This study
pJET-D3	1482 bp AFase-D3 gene amplicon blunt-end ligated into pJet1.2	This study
pET21a(+)	Expression vector with a C- terminal His-tag, AmpR, T7 promoter and terminator, MCS.	Novagen, USA
pET21a-H4	1467 bp NdeI-XhoI fragment from pJET-H4 cloned in pET21a.	This study
pET21a-E3	1547 bp NdeI- HindIII fragment from pJET-H4 cloned in pET21a.	This study
pET21a-D3	1482 bp NdeI-XhoI fragment from pJET-H4 cloned in pET21a.	This study

^a^ Plasmid encoding Ampicillin and Chloramphenicol resistance is indicated as Amp^R^ and Cam^R^, respectively. Ori is used as an abbreviation for origin of replication and MCS is the acronym for multiple cloning site. The Hexa-histidine tag is abbreviated to His-tag that was used to purify the AFase proteins of this study through nickel-ion affinity liquid chromatography

**Table 2:** Primers used in this study

Primer	5′ to 3′ sequence^a^	Reference
T7 Promoter forward	TAATACGACTCACTATAGGG	Epicentre® Biotechnologies
pCC1Fos reverse	CTCGTATGTTGTGTGGAATTGTGAGC	Epicentre® Biotechnology
MUKAN-1 FP-1	CTGGTCCACCTACAACAAAGG	Epicentre® Biotechnologies
MUKAN-1 RP-1	AGAGATTTTGAGACAGGATCCG	Epicentre® Biotechnologies
M13 Forward	CCCAGTCACGACGTTGTAAAACG	Inqaba Biotec
M13 Reverse	AGCGGATAACAATTTCACACAGG	Inqaba Biotec
H4-pET-Fwd	GTT**CATATG**AATCACATCAAGATTGATTTAGAT	This Study
H4-pET-Rev	CG**CTCGAG**TAAGTCAAAGCTGAGC	This Study
E3-pET-Fwd	AT**CATATG**GACGGAGGCGCATGCG	This Study
E3-pET-Rev	C**AAGCTT**GGACGGTCGGCGG	This Study
D3-pET-Fwd	GAT**CATATG**AACAATGTCGTCATCAATGTGG	This Study
D3-pET-Rev	CTT**CTCGAG**ACCTAATCTTAGAATGCCGAC	This Study

^a^The restriction endonuclease sites incorporated into the PCR primers include *Xho*I, *Nde*I and *Hin*dIII indicated as bold nucleotides

### Fosmid selection

AFase positive clones identified during primary screening in [15] were first subjected to thermostability assays to identify those with desirable properties. Briefly, *E. coli* harbouring recombinant fosmids were inoculated into microtiter plates with LB containing 0.01% (*w*/*v*) L-arabinose and 15 μg/mL chloramphenicol and incubated for 16 h. A 200 μL aliquot of the overnight cultures was lysed with 10 μL Bugbuster™ protein extraction reagent (Novagen, USA) and the extracts incubated at 25, 40, 50, 60, 70, 80 or 90 °C for 60 min. Fosmid cell-free extracts incubated on ice served as controls. After incubation, the thermally treated extracts were placed on ice for 30 min before centrifugation at 13000 x g for 5 min at 4 °C to remove cell debris. Aliquots of 100 μL were then transferred into a flat bottom 96 well microtiter plate (Sterilin®) and *p*NP-arabinofuranose in 100 mM NaPO_4_ (pH 7.0) was added to the extracts at a final concentration of 2 mM. The microtiter plate was incubated at 37 °C for 60 min and AFase activity was measured using a spectrophotometer at 410 nm (SPECTROstar Nano; BMG Labtech). All assays were performed in triplicate. From these assays, pFos-H4, pFos-E3, and pFos-D3, were identified as expressing the most thermostable AFase activities and selected for further study.

### Transposon mutagenesis for identification of AFase encoding open reading frames

Transposon mutagenesis was performed using the HyperMu™ < KAN-1 > Insertion Kit (Epicentre® Biotechnologies, USA) according to the manufacturer’s instructions. Thereafter, the transposon-treated fosmid library together with respective control fosmids lacking the AFase insert were transformed into electrocompetent *E. coli* Epi300 cells and cultured on LA containing 15 μg/mL chloramphenicol and 50 μg/mL kanamycin. Single colonies growing on double selection plates were sub-cultured onto LA medium supplemented with chloramphenicol and kanamycin and cultured for 16 h at 37 °C. Subsequently, the mutated fosmid library and the respective controls were inoculated into individual wells of 96-well microtitre plates (Sterilin®) containing LB supplemented with kanamycin and chloramphenicol. The microtitre plates were duplicated using a 96-pin QPix2 automated colony picker (Genetix) and were incubated as described above. Thereafter, the cells were lysed to release soluble cell-free extract using BugBuster™ protein extraction reagent (Novagen). Enzyme activities of the mutants and their respective untreated AFase controls were detected by the addition of 1 mM *p*NP-arabinofuranose in 50 mM NaPO_4_ buffer (pH 7.0) to each well of the microtiter plate containing the cell-free extracts and incubated at 37 °C until the development of a yellow colour indicating the release of *p*NP from the synthetic substrate. Transposon-treated fosmid clones that did not develop the distinct yellow colour or were observed to produce significantly reduced levels of yellow colour relative to that of the untreated fosmid controls were identified and chosen for further characterization.

Sequencing of mutated fosmids was conducted by the University of Stellenbosch’s Central Analytical Facility (CAF). The sequences were manually edited using Chromas version 2.01 (Technelysium DNA sequencing software, Australia) and DNAMAN version 4.13 (Lynnon Corp., San Ramon, CA, USA). Sequence identity and similarity searches of DNA sequences were performed using the basic local alignment search tool (BLAST) programs as provided by the National Centre for Biotechnology Information (NCBI) [16, 17]. Putative ORFs were identified within the consensus sequence using GeneMark for prokaryotes (https://tinyurl.com/ya78gkes) and Interproscan [18] was used to identify conserved protein domains. The gene sequences for the three AFases were submitted to the GenBank database with accession numbers (QBG80847-QBG80849).

### Expression and purification of AFases

The AFase genes, AFase-H4, AFase-E3 and AFase-D3 were amplified from the purified fosmid DNA with each primer bearing a specific restriction site for cloning into pET vectors (Table 1). The polymerase chain reactions (PCRs) were performed using Phusion DNA polymerase (ThermoFisher Scientific™) with AFase-H4 and AFase-D3 genes amplified from the fosmid DNA using the following conditions; initial denaturation at 98 °C for 30 s followed by 35 cycles of 10 s at 98 °C for denaturation, 30 s at 70 °C for annealing, 45 s at 72 °C for elongation and the final elongation at 72 °C for 10 min. Similar conditions were employed for amplifying the AFase-E3 gene except for annealing and elongation times which were reduced to 15 s and 30 s, respectively. The resulting PCR products were purified using the gel extraction kit from Machery Nagel. These were cloned into the pJET1.2/blunt cloning vector to create the recombinant constructs pJET1.2-AFase-H4, pJET1.2-AFase-D3 and pJET1.2-AFase-E3. The cloned inserts were sequenced to confirm that PCR errors were not introduced. The cloned genes were subsequently excised from the recombinant pJET-AFase constructs and cloned into the pET21a (+) vector digested with the restriction enzymes engineered into the primer sequences (Table 2). Following plasmid miniprep, the DNA sequence of constructs observed to contain the correct sized inserts was determined to ensure that the putative AFase genes had been cloned in-frame with the promoter and C-terminal histidine tag. The recombinant constructs, pET21a-H4, pET21a-E3 and pET21a-D3 were transformed into chemical competent *E. coli*-BL21 (DE3) (Table 1) cells for protein expression.

Two millilitres from overnight starter cultures of *E. coli* harbouring the respective pET21 constructs were inoculated in 500 mL Erlenmeyer flasks containing 50 mL LB with appropriate antibiotics and incubated to an OD_600nm_ of approximately 0.6–0.8. Protein expression was induced by treating two experimental cultures with 0.5 and 1 mM isopropyl β-D-1-thiogalactopyranoside (IPTG, Fermentas) respectively. The third culture served as an uninduced control. Following incubation for three hours all the cultures were centrifuged at 4000 x g for 20 min at 4 °C. The pellets were resuspended in 10 mL of 1 x binding buffer (250 mM NaCl, 20 mM Tris-HCl, 5 mM imidazole, pH 7.9) and then disrupted by sonication (Bandelin Sono plus Ultrasonic Homogenizer, Germany) on ice with 6 pulses of 20 s. Following sonication, the lysates were centrifuged at 6000 x g for 20 min at 4 °C to remove cell debris. Proteins were purified by nickel affinity chromatography using the His-Bind® resin and buffer kit (Novagen, USA) following the manufacturer’s instructions and dialysed against 200 volumes 20 mM sodium phosphate; 50 mM sodium chloride buffer (pH 7.0) using a 10 kDa MW cut-off membrane (Thermo Fischer Scientific, USA) for 16 h at 4 °C. The concentrations of purified AFases were determined by the method of Bradford [19] using the Bio-Rad protein assay kit with bovine serum albumin as a standard. Protein purity was assessed by sodium dodecyl sulphate-polyacrylamide gel electrophoresis (SDS-PAGE) under denaturing conditions as described by Laemmli [20].

### Biochemical characterization

Thermal stability assays with purified AFases were performed by incubating them for 90 min or 24 h at various temperatures (25, 40, 50, 60, 70, 80 and 90 °C). For the AFases incubated for 24 h, samples were removed at 2 h intervals until 12 h and at 24 h. AFase activities were measured as described for cell-free thermal stability assays in preliminary screening described above with the respective control reactions incubated on ice.

The optimum temperature of the purified AFases (0.5 μg per assay) was assessed by measuring the activity of each AFase against 2 mM *p*NP-arabinofuranosyl at 6, 20.5, 25, 40, 50, 60, 70, 80 and 90 °C after 2 min of incubation with the substrate at the indicated temperatures. The temperature at which the highest activity was recorded was selected as the optimum temperature for each AFase tested. The optimal pH of the AFases was evaluated at 40 °C in 50 mM citrate and/or phosphate buffers with pH varying from 2 to 8 and the buffers without AFase added were used in control reactions. The pH of the buffer containing the highest AFase activity was selected as the optimal pH for each AFase tested.

### Substrate specificity and enzyme kinetics

For substrate specificity the following substrates were assayed: *p*NP-β-D-cellobioside, *p*NP-α-D-mannopyranoside, *p*NP-β-D-fucopyranoside, *p*NP-β-D-glucuronide, *p*NP-α-D-glucopyranoside, *p*-NP- β-D-glucopyranoside *p*NP-α-L-arabinopyranoside and *p*NP-β-L-arabinopyranoside from Sigma-Aldrich (SA) and *p*NP-α-L-arabinofuranoside, *p*NP-β-D-xylopyranoside, *p*-methylumbelliferyl-β-D-xylopyranoside and *p*NP-β-D-mannopyranoside from Carbosynth Ltd. All the substrates were prepared following their respective manufacturer’s instructions. The enzymatic assays were performed in a total reaction volume of 250 μL containing 2 mM substrate in 50 mM citrate buffer (pH 7.0) with 0.5 μg enzyme for 2 min. The reactions were stopped by raising the pH with the addition of 750 μL of 0.4 M Na_2_CO_3._ A 250 μL reaction volume was aliquoted into a single well of a 96-well microtiter plate (Sterilin®). The enzyme activities were measured spectrophotometrically at 410 nm and the standard curve was generated using 1–10 mM pNP under assay conditions. One unit (U) of the enzyme is defined as the amount of enzyme that can liberate 1 μmol of *p*NP per minute. All assays were performed in triplicate with the addition of the appropriate controls. Complex substrates were hydrolyzed for four hours at each enzyme’s apparent temperature optimum and pH. Reducing sugars resulting from the hydrolysis of complex α-L-arabinose polysaccharide substrates: arabinoxylan, arabinan and linear arabinan (Megazyme, 0.5% *w*/*v*) were measured using the dinitrosalicylic acid (DNS) assay as described by Miller [21]. Briefly, 150 μL of DNS solution was added to 50 μL reactions and boiled for 5 min before being rapidly cooled on ice. The volume was made up to 1000 μL with dH_2_O and the absorbance determined at 510 nm (OD510; SPECTROstar Nano; BMG Labtech). The reducing sugars were determined using a L-arabinose standard curve generated under the same reaction conditions. Kinetic parameters (*K*_*M*_, *V*_*max*_ and *k*_*cat*_) were evaluated by measuring the enzyme activity in the presence of varying *p*NP-arabinofuranosyl concentrations after 1 min. The Michealis-Menten plot was generated using GraphPad Prism 4 (GraphPad Software).

### Structural analysis of AFases

Prediction of the quaternary structures was performed by fast protein liquid chromatography (AKTA FPLC, Amersham Biosciences) using a Superdex G200 column run at 0.5 ml/min with running buffer (50 mM NaCl, 20 mM Na_2_PO_4_ (pH 7.0)). The proteins were resuspended in running buffer and loaded onto the column according to the manufactures instructions. Proteins used as molecular weight markers were β-amylase (200 kDa), alcohol dehydrogenase (150 kDa), albumin (66 kDa) and carbonic anhydrase (29 kDa) which were also resuspended in the same running buffer. These were used to plot the log of their MW against retention time to generate a standard curve. Protein molecular weights were approximated based on the retention time using the standard curve generated.

## Results

### Functional screening and selection of AFase encoding fosmids

In a previous study by Ohlhoff et al. [14], a metagenomic library consisting of more than 150,000 fosmid clones with an average insert size of 31 kb was constructed. A total of 46,000 clones were screened for α-L-arabinofuranosidase (AFase) activity and 13 putative AFase positive clones were identified [15]. Here we performed preliminary thermostability screening by incubating cell-free extracts at a range of temperatures for 60 min. Cell-free extract from pFos-D3 AFase retained close to 100% activity when incubated at 25 and 40 °C. The enzyme activity decreased by approximately 40 and 90% when incubated at 50 and 60 °C, respectively, while at 70 °C and higher, negligible AFase activity was detected. AFase pFos-H4 retained 100% activity following incubation at 25, 40, 50 and 60 °C. The relative residual activity decreased sharply following incubation at temperatures greater than 60 °C. In contrast to pFos-D3, the AFase activity encoded by cell-free extract from pFos-H4 and pFos-E3 displayed an increase in relative activity following incubation at temperatures greater than 30 °C, with the highest activity observed between 60 and 70 °C respectively. At these temperatures, the recombinant AFase-E3 maintained full activity, relative to the untreated control, and was the most thermostable of the three enzymes. Based on these preliminary thermostability profiles, the crude protein extracts of pFos-D3, pFos-H4 and pFos-E3 clones were identified as thermolabile, moderately thermostable and thermostable respectively, and were selected for further characterization.

### Sequence and phylogenetic analysis of AFase-encoding ORFs

Transposon mutagenesis was employed to identify the ORFs encoding the α-L-arabinofuranosidase activities. Fosmids with loss of, or decreased activity, compared to the non-mutated constructs, indicative of gene disruption, were selected for sequence analysis. Overlapping sequences were generated from 4 to 6 mutants for each fosmid (pFos-H4, pFos-E3 and pFos-D3) to assemble putative AFase encoding regions of 2693, 2406 and 2505 bp for each fosmid, respectively.

The putative ORF (489 amino acids; 55 kDa) from pFos-H4 shared 100% amino acid identity with a sequence derived from a Brazilian compost metagenome (QGUR01000024.1) which, although reported on earlier [22], was only recently added to the NCBI database (June 2018). One of the pFos-H4 end sequences also matched 100% at nucleotide level to the metagenomic contig this gene was located on, suggesting that AFase-H4 originates from a closely related bacterium. The next closest hit was to an AFase from *Truepera radiovictrix* DSM 17093 at 61% identity. Conserved catalytic and C-terminal domains were identified within the predicted AFase amino acid sequence, and no evidence of a signal peptide was detected. This could indicate that a “non-classical protein secretion” mechanism may be involved in exporting the AFase-H4 protein [23]. The catalytic domain overlaps the AFase C-terminal domain and is positioned from P41-A346 and E261-V482, respectively. A putative cellulase-like domain is located within the catalytic domain from V161-W240. The catalytic domain has sequence identity to that of Glycoside Hydrolase (GH) family 51 (GH51).

For pFos-E3, the predicted ORF consists of 510 amino acids (58 kDa) and the sequence shared 99% identity over the full length of the protein to a putative AFase from *Paenibacillaceae* bacterium JTherm, also isolated from a compost heap in Vacaville, CA, USA [24]. Nucleotide sequence from both ends of pFos-E3 also matched 100% at the nucleotide level to this genome, indicating that AFase-E3 likely originates from an identical or very closely related bacterium. This organism has been proposed to be the first member of a new genus *Candidatus Reconcilibacillus cellulovorans* responsible for the initial breakdown of cellulosic material in a compost heap allowing other community members to proliferate and hydrolyse the material further [24].

The 494 amino acid (56 kDa) sequence of pFos-D3 shared 77% identity (87% similarity) with a *Paenibacillus taihuensis* ORF. Although not yet published, this organism’s genome was sequenced as part of the third phase of the Joint Genome Institutes’ Genomic Encyclopedia of Type Strains program which covers genomes from soil and those that are plant associated. It could be argued that these are mesophilic environments and may be the reason AFase-D3 displays mesophilic characteristics.

All the key amino acids in the catalytic site of AFases have previously been identified [25] and these were perfectly conserved in both AFase-H4 and AFase-E3 (F23, E25, R65, N70, W95, N170, E171, H239, Y241, E289, W293, and Q340; AFase-H4 numbering). In AFase-D3 however, Asn70 is replaced by a cysteine residue. This residue is thought to be important to substrate binding as a hydrogen bonding partner to Glu25. Glu25 may not be directly involved in catalysis but is thought to be crucial in coordinating the hydrogen bonding network at the non-reducing end of the substrate and in this way enable substrate binding [26]. Despite this substitution the enzyme is still functional and this may be part of the reason why a broad apparent temperature optimum is observed as explained in the discussion.

**Fig. 1:**
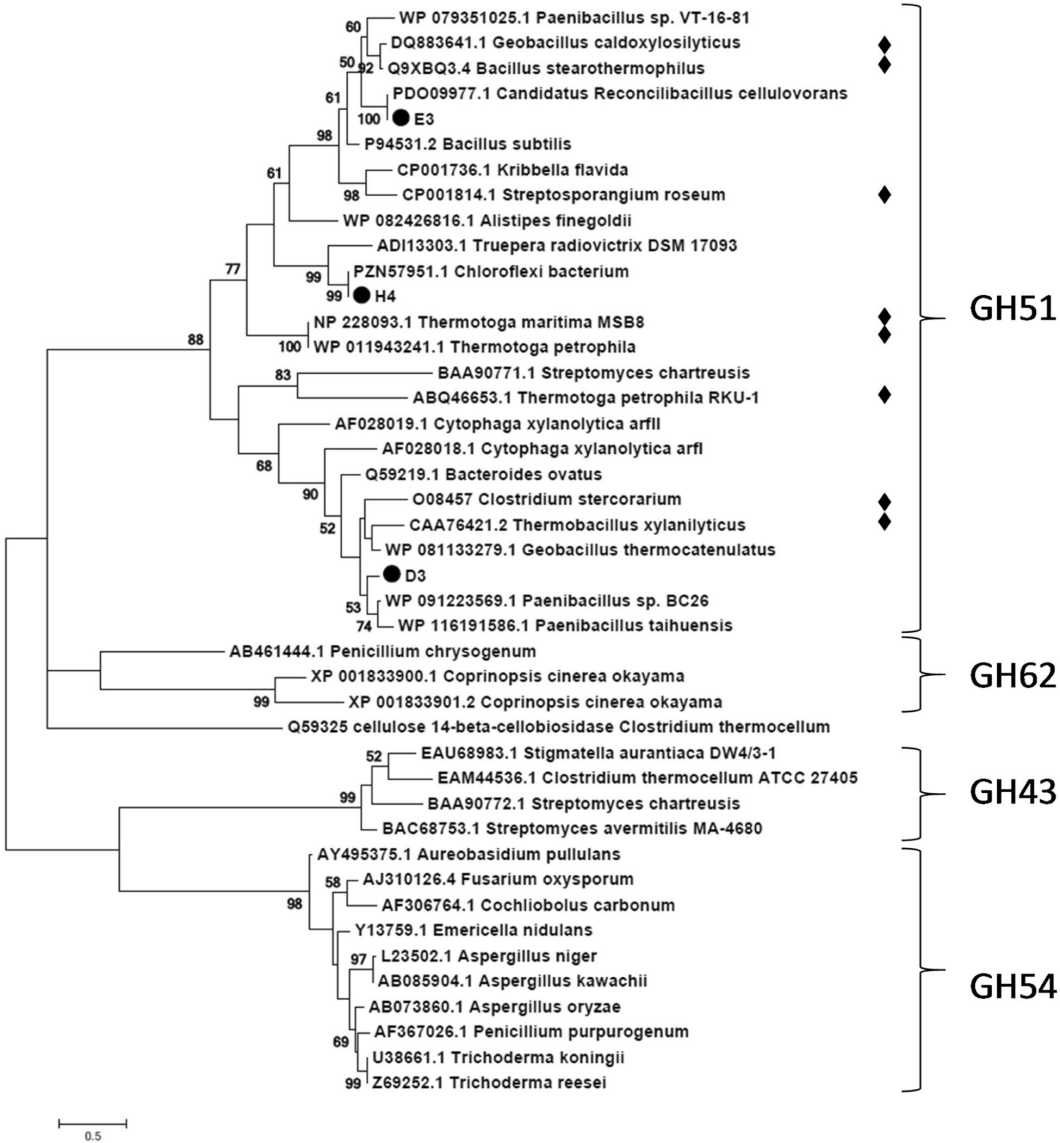
Maximum Likelihood (JTT model) tree of the phylogenetic relationship of AFase-H4, −E3 and -D3 to characterised AFases from four glycoside hydrolase families. The functionally characterized thermophilic AFases are indicated with the black diamond symbol (♦). The scale bar indicates the number of amino acid substitutions

Phylogenetic analysis of the three AFase sequences showed that they all clustered together with representative AFase sequences of the GH51 family which contains several thermostable proteins (Fig. 1). The sequences of the more thermostable AFase-H4 and AFase-E3 are more related as compared to the less thermostable AFase-D3 (Fig. 1). Both AFase-E3 and AFase-D3 show similarity to AFases from *Bacillus subtilis*, a group that contains both mesophilic and thermophilic variants of this protein [27, 28].

### Biochemical characterization of the recombinant proteins

The three selected AFase ORFs were cloned and expressed in *E. coli* and purified to near homogeneity as judged by SDS-PAGE analysis using nickel-ion affinity chromatography (results not shown). All three proteins showed molecular masses close to their in silico predicted sizes of 55 kDa, 56 kDa and 58 kDa for AFase-H4, −D3 and -E3 respectively. The quaternary structures of the native AFase-H4, AFase-E3 and AFase-D3 proteins were determined by gel filtration. The respective retention times of the three AFases, relative to that of molecular weight standards suggested that all three proteins are homo-hexamers or a trimer of dimers.

**Fig. 2:**
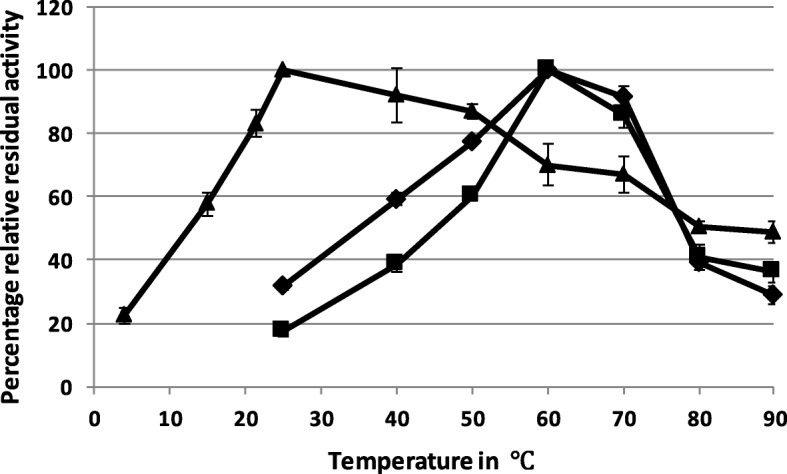
The temperature optima of AFase H4 (♦), E3 (■) and D3 (▲). Data represents the mean ± standard error (*n* = 3)

**Fig. 3:**
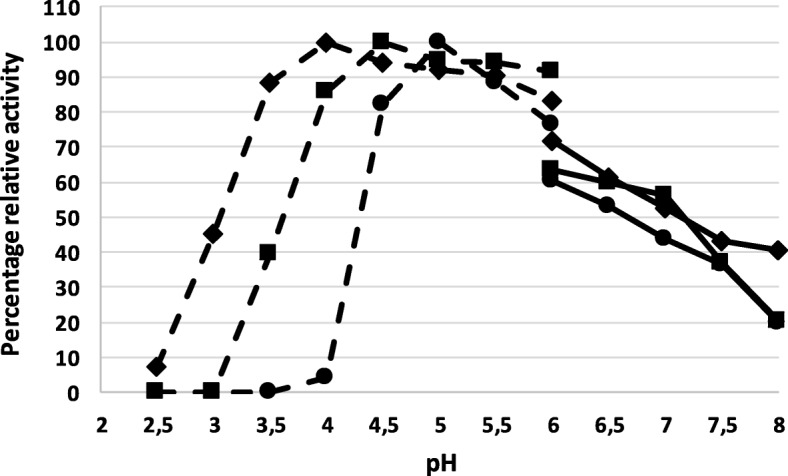
pH optima of AFase H4 (●), E3 (♦) and D3 (■) determined in phosphate (dashed line) and citrate buffers (dotted line) between pH 2.5 and 6.0 and pH 6.0 to 8.0, respectively. Data represents the mean ± standard error (*n* = 3)

AFase-H4 and AFase-E3 had an apparent temperature optimum of 60 °C while for AFase-D3 the apparent optimal temperature was 25 °C (Fig. 2). Thermal stability analysis showed that AFase-H4 maintained 100% residual activity even after a prolonged incubation time of 12 h at 50 °C. The residual activity started to decrease when the protein was incubated at 60 °C. AFase-D3 maintained 100% residual activity after incubation for 1 h at 40 °C, whereas AFases H4 and E3 displayed classic temperature dependence profiles. Interestingly, AFase-D3 maintained approximately 50% activity even at 80–90 °C. AFase-E3 maintained 100% residual activity even after incubation for 24 h at 70 °C, the most thermostable of the three enzymes identified (Additional file 1: Figure S1). All three AFases showed best activity in the pH range 4.0–6.0 (Fig. 3).

**Fig. 4:**
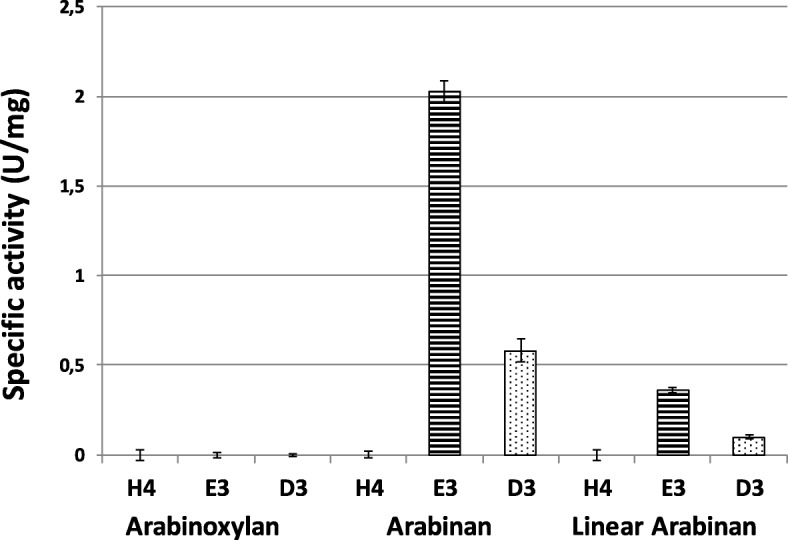
AFase activity on complex substrates, namely arabinoxylan, arabinan and linear arabinan. The specific activity was determined in U/mg. (*n* = 3) ± standard error

Due to the various domains identified for these enzymes based on sequence analysis, the substrate specificities of these AFases were assessed on various pNP-linked substrates. All three AFases showed strong activity towards pNP-α-L-arabinofuranoside, with AFase-E3 displaying the highest activity at about 130 U/mg, followed by AFase-D3 (~ 90 U/mg) and AFase-H4 (~ 50 U/mg) (Additional file 2: Figure S2). AFase-H4 also showed substantial activity on pNP-β-D-cellobiose (~ 10 U/mg) while relatively low but detectable activities were displayed by all AFases when assayed on other pNP-linked substrates. However, none of the AFases were able to hydrolyse pNP-β-D-xylopyranoside substrate. The hydrolytic preferences of these enzymes were also examined on more complex substrates including arabinoxylan, arabinan, and linear arabinan (Fig. 4). These polysaccharide substrates were chosen to represent differing internal bonds between arabinose subunits or sidechains. All three AFases displayed different effectiveness for hydrolysing arabinofuranosyl bonds within these three substrates. AFase-E3 showed highest activity on these substrates (arabinan and linear arabinan) when compared to AFase-D3 while AFase-H4 was incapable of hydrolyzing any of these substrates and none of the AFases released reducing sugars from arabinoxylan.

All three enzymes displayed Michaelis-Menten kinetics when assayed on pNP-α-L-arabinofuranoside (Additional file 3: Figure S3). AFase-H4 and AFase-E3 displayed slightly lower K_*M*_ values when compared to AFase-D3 indicating that AFase-D3 has a slightly lower affinity for this substrate compared to the other two enzymes. AFase-E3 showed the highest specific activity and the highest turnover number of the three enzymes (Table 3).

**Table 3:** Enzyme kinetic comparison of AFases characterised in this study and thermostable AFases obtained from literature

AFase	Micro-organism	K_*M*_ in mM (Std. error)	Sp. act. in U/mg (Std. error)	k_*cat*_ (s^−1^)	k_*cat*_/K_*M*_ (M^−1^ s^−1^)	Reference
*AFase-H4*	Unknown	0.31 (±0.03)	143.1 (±3.7)	131	4.2 × 10^5^	This study
*AFase-E3*	Unknown	0.33 (±0.04)	228.6 (±9.7)	219	6.6 × 10^5^	This study
*AFase-D3*	Unknown	0.38 (±0.05)	166.6 (±6.9)	155	4.1 × 10^5^	This study
abf51S9	*Streptomyces* sp. S9	1.45	221	203	1.4 × 10^5^	[29]
AbfATK4	*Geobacillus caldoxylolyticus* TK4	0.17	588	568	3.3 × 10^6^	[30]
AbfAC26Sari	*Anoxybacillus kestanbolensis*	0.14	1019	968	7 × 10^6^	[31]
Tm-Afase	*Thermotoga maritima*	0.42	23.5	22	5.2 × 10^4^	[42]
AFase	*Caldocellulosiruptor sacchrolyticus*	1.29	295	285	2.2 × 10^5^	[60]
abfB	*Auriobasidium pullulans*	6.27	78.1	96.3	1.5 × 10^4^	[62]

## Discussion

Here we present three novel AFases, identified from a compost metagenomic library. Although all three sequences have close relatives on the GenBank database, owing to the recent addition of metagenomic and bacterial genome sequence, none of these closely related enzymes have been characterized previously. The presence of nearly identical AFase-H4 and AFase-E3 sequences in compost heaps that are biogeographically separated speaks to them being selected for and particularly successful in compost environments. All bioinformatic and biochemical characterization data presented supports the classification of these three enzymes as belonging to the GH51 family [EC 3.2.1.55]. The AFases shared a homo-hexameric structure or possibly consist of a dimer of trimers. Although hexameric structures for AFase proteins have been reported, specifically for the GH51 family of AFases, tetramers and octamers have also been reported from this family. Homo-tetrameric structures have been reported for enzymes from a *Streptomyces* species, *Geobacillus caldoxylolyticus* TK4 and *Anoxybacillus kestanbolensis* AC26Sari [29–31].

AFase-H4 and AFase-E3 displayed optimal activity at 60 °C while AFase-D3 displayed a mesophilic thermal profile with an optimum activity at 25 °C yet retaining up to 50% activity at temperatures as high as 80–90 °C. The thermostability profiles of AFase-H4 and AFase-E3 were both similar to that of functionally characterized AFases from *Streptomyces* sp. S9 [29], *Paenibacillus* sp*.* TH51 [7] and *G. caldoxylolyticus* TK4 [30]. AFase-E3 shares sequence similarity with *B. subtilis* derived AFase and this species is known to encode both mesophilic and thermophilic AFases. The presence of AFases with both mesophilic and thermophilic properties in compost could be explained by the stages involved during the composting process. In the early stages of composting mesophilic microorganisms dominate and their metabolic activities cause an increase in temperature. This stimulates the growth and activity of thermophilic microorganisms which is subsequently followed by a cooling stage also referred to as the maturation stage [32, 33]. Since the AFases described here are derived from compost that reaches only 70 °C, this may put an upper limit on the thermal stability and apparent temperature optimum of enzymes we could have identified in this study.

The mesophilic/thermophilic characteristic displayed by AFase-D3 has only been reported for AFases that belong to the GH43 family previously isolated from *Paenibacillus* species [34] with no characterized GH51 family enzyme displaying this thermal profile. Broad temperature optima have been observed for several different enzymes isolated from a range of organisms including plants [35–38]. Of particular interest are enzymes (phophoribosyl anthranilate isomerase, indoleglycerol phosphate synthase and l-isoaspartyl (D-aspatyl) O-methyltransferase) from *T. maritima* that show highest catalytic efficiency at 25 °C as opposed to the organism’s optimum growth temperature [39–41]. *T. maritima* has a wide growth temperature range up to its optimum of 90 °C. Although a thermally robust GH51 family AFase which could maintain full activity even at temperatures as high as 90 °C has been isolated from *T. maritima* MSB8, there was no evidence of a thermophilic/mesophilic property for this enzyme [42]. While the thermal behavior of AFase-H4 and AFase-E3 correspond to enzymes which have a large Δ*H*_*eq*_ for the E_act_/E_inact_ transition according to the equilibrium model for dependence of enzyme activity on temperature, AFase-D3 likely has a small Δ*H*_*eq*_ resulting in the broad temperature optimum [43]. A small Δ*H*_*eq*_ may be the result of the substrate used to assay for the apparent temperature optimum as the E_act_/E_inact_ transition appears to be linked to the active site conformation. It could be that the *p*NP-arabinofuranosyl substrate locks the active site, and by extension other parts of the protein, in a stable conformation. Taken together, this leaves open the possibility that AFase-D3 is a thermophilic enzyme. The *Thermotogae* phylum contains microorganisms with temperature ranges that span both thermophilic to mesophilic temperatures and they are interesting models for studying evolutionary changes [39, 44]. This flexibility has been ascribed to lateral gene transfer where most of the acquired genes in this phylum are involved in carbohydrate metabolism [44]. These gene acquisitions afford the bacteria in this phylum a selective advantage to transit between the mesophilic and thermophilic environments and of metabolizing across a broad spectrum of temperatures [45, 46]. It would be of interest to isolate the host of the AFase-D3 to assess whether the thermostability profile observed for this protein extends to the entire organism as an adaptation mechanism as described for *T. maritima* and other *Thermotogae* lineages.

Studies conducted to discriminate between mesophilic and thermophilic proteins have shown that there are no significant differences in these protein orthologues as they often share a similar structure, sequence identity and the same catalytic mechanisms [45]. There appears to be no uniform property that confers increased protein thermostability across the proteome, but rather could be a result of minor differences in sequence and structure due to point mutations, increased numbers of salt bridges and the presence of specific amino acid residues on the protein surface such as fewer thermolabile amino acid residues observed in thermophilic proteins [46]. This seems to be evident in this study as cysteine residues which are often associated with increased protein thermostability were identified in the protein sequence of AFase-E3, which showed the highest thermostability among the AFases described in this study. Cysteine residues are known to form disulphide bridges with alternate cysteine residues within the catalytic domain and to increase thermostability by 10 to 20 °C [29, 47]. The contribution of cysteine residues to the thermostability has been previously shown through substitution of cysteine residues with alanine, resulting in a decreased thermostability of AFases from *G. caldoxylolyticus*, *Geobacillus stearothermophilus*, *Thermobacillus xylanilyticus* and *B. subtilis* [30, 48]. Further evidence of the contribution of subtle changes in the amino acid sequence to thermostability of the proteins has been shown through comparison of amino acid composition of thermophilic and mesophilic protein homologues. The diguanylate cyclase and glutamate dehydrogenase enzymes from the hyperthermal *T. maritima* had a smaller hydrophobic accessible surface and a greater charged surface area when compared to their mesophilic homologues from *Pseudomonas aeruginosa* and *Clostridium symbiosum*, respectively [44]. This conformation results in stronger hydrophobic interactions in the interior of the protein and increased ion pairing on the surface conferring greater thermostability to these thermophilic proteins compared to their respective mesophilic counterpart [45].

The three AFases described here had optimum activity within a slightly acidic pH range of 4.0–5.0. A pH optimum in this range is typical for AFases belonging to the GH51 family as previously shown for AFases from *Paenibacillus* sp., *Aureobasidium pullulans*, *Aspergillus oryzae* and *Streptomyces coelicolo*r [7, 49]. Industrial processes often operate at high temperatures and slightly acidic pH which make the AFases identified in this study suitable for application processes conducted under these conditions [50].

All three AFases displayed the greatest hydrolytic activity towards pNP-α-L-arabinofuranoside compared to the activity on the other pNP-glycosides. Of the 145 GH families reported to date only families GH2, 3, 43, 51, 54 and 62 are known to encode α-L-arabinofuranosidase activity with family GH51 having the largest number of α-L-arabinofuranosidases [6, 51]. Lower activity was observed on the other pNP-glycosides except for AFase-H4 which showed substantial activity on pNP-β-D-cellobioside. The activity on pNP-β-D-cellobioside shown by AFase-H4 is the first report for a GH51 family AFase. None of the AFases could hydrolyse the pNP-β-D-xylopyranoside substrate. The enzymatic hydrolysis of pNP-β-D-xylopyranoside is a characteristic of the GH43 family of AFases, which are known for their dual activity, possessing both β-xylosidase and the conventional α-L-arabinofuranosidase activity [52]. Only two GH51 AFases have been shown to hydrolyse this bond and these were isolated from *G. caldoxylolyticus* TK4 [30] and *Paenibacillus sp*. TH51 [7]. AFase-D3 had very low activity on pNP-β-D-glucopyranoside and pNP-α-L-mannopyranose. No other GH51 AFase has been reported to have the capability of hydrolyzing these synthetic substrates and together with its exceptionally wide thermostability profile, it makes it a unique enzyme.

All three AFases showed different levels of activity for hydrolysis of the arabinofuranosyl bonds within the natural substrates tested. AFase-E3 indicated a higher affinity for these substrates when compared to AFase-H4 and AFase-D3. The hydrolysis of arabinan has been a common functional characteristic for GH51 AFases [53] but, AFase-H4 showed no hydrolysis of arabinan. Similarly, the AFases from *Streptomyces* sp. and *Penicillium purpurogenum* were unable to hydrolyse this substrate [54, 55]. AFase-E3 and AFase-D3 had a lower activity on linearized arabinan and it is known that GH51 AFases weakly hydrolyze the α-1,5-L arabinofuranosyl bonds, whereas this is a capability of the GH43 family AFases [52, 53]. A similar linear arabinan hydrolysis profile has been reported previously [54] suggesting that the AFases described here are typically exo-acting enzymes contrary to GH43 AFases which readily hydrolyze the α-1,5-L arabinofuranosyl bonds [34, 52]. None of the AFases were able to release reducing sugars from arabinoxylan. This activity is characteristic of some GH51 AFases, but not all [29–31, 47, 56]. Substrate specificity towards arabinoxylan has been attributed to specific residues in the catalytic domain in the *Thermotoga* species and *Thermobacillus* species of AFases [47, 57]. A tryptophan located at approximately the 96-100th position in the catalytic domain [47, 57] is a key xylan-binding residue. However only AFase-E3 has two of these residues required for an electrostatic active site to liberate L-arabinose from the xylan backbone. Figure 3.20 shows no liberation of L-arabinose from the xylan backbone and this could be due to the absence of the residues required for an adequate electrostatic surface within the active site [26]. The inability to liberate the L-arabinose from the xylan backbone has also been correlated to the ratio of the α-1,3-L and α-1,2-L- arabinofuranosyl bonds that differs within different arabinoxylan-containing materials. There are particular arabinoxylans that are highly saturated with either α-1,3-L or the α-1,2-L bonds [58]. Therefore, it is plausible that the arabinoxylan used in this experiment is saturated with more of α-1,2-L bonds. This conclusion is consistent owing to the AFases showing an affinity for the *p*-nitrophenyl-α-L-arabinofuranoside bond which resembles the α-1,3-L arabinofuranosyl bonds [58, 59].

Multifunctional enzymes with two or more activities are highly desirable for the hydrolysis of complex polymers and offer the possibility of reduced complexity of enzyme cocktails and better synergies [7]. However, as evidenced in this study, the shortfall is that these additional activities are always minor and are not displayed when GH51 AFases are employed to hydrolyse heteroxylan or xylo-oligosaccharides in more complex and natural substrates [7]. The relatively good cellobioside activity of AFase-H4, together with its moderate thermostability, could make this enzyme a good starting point for engineering of a dual functional enzyme for use in thermophilic ethanologenesis processes.

Although we have highlighted the unique features and differences that exist between these and previously described enzymes, overall, the kinetic data for these three AFases demonstrate that they are not exceptional in this regard and have characteristics that are in the same range to that of previously characterized AFases [30, 31, 60]*.* Thus, even though these AFases are phylogenetically distinct, their biochemical characteristics are similar to those that have been described previously. This recurring theme in enzymology, once again reiterates the power of selective pressure to ensure that the enzymes’ activity falls within a limited range [61]. However, the hydrolysis of bonds other than the α-1,5-L-arabinofuranosyl linkage makes the AFases identified in this study novel and they could be used as a starting point for engineering to optimise their suitability for various industrial applications.

## Conclusions

Here we describe three GH51-related α-arabinofuranosidases that are novel at the amino acid level, substrate specificity and thermostability profile (AFase-D3) with the potential to be engineered to be used in industrial processes. Their discovery once again demonstrates the power of functional metagenomics and evolutionary pressure to explore novel sequence space.

## Additional files

Additional file 1: **Figure S1.** Thermostability profiles for the purified AFase H4 (A), E3 (B) and D3 (C) at 25 (✚), 40 (▲), 50 (♦), 60 (■), 70 (╳) and 80 °C (●). Data represents the average of three replicates ± standard error (*n* = 3). (DOCX 30 kb)

Additional file 2: **Figure S2.** Substrate range of AFases measured on pNP linked glycosides. A) H4, B) E3 and C) D3. Data represents the average of three replicates ± standard error (*n* = 3). (DOCX 46 kb)

Additional file 3: **Figure S3.** Michaelis-Menten plots of AFase-D3, −E3 and -H4. The kinetics of each enzyme was determined at their respective pH and temperature optima with increasing amounts of pNP-α-L-arabinofuranoside (mM). (*n* = 3) ± standard error. (DOCX 72 kb)

## Abbreviations

AFases: Arabinofuranosidase
GH: Glycosyl hydrolases
IPTG: Isopropyl-β-D-1-thiogalactopyranoside
NGS: Next generation sequencing
ORF: Open reading frame
*p*NP: *para*-nitrophenyl
