# Supplementary figures and images for: Characterisation of three novel α-L-arabinofuranosidases from a compost metagenome
Source: BMC Biotechnol. 2019 Apr 18;19:22. doi: 10.1186/s12896-019-0510-1 (PMC6472066; doi:10.1186/s12896-019-0510-1)

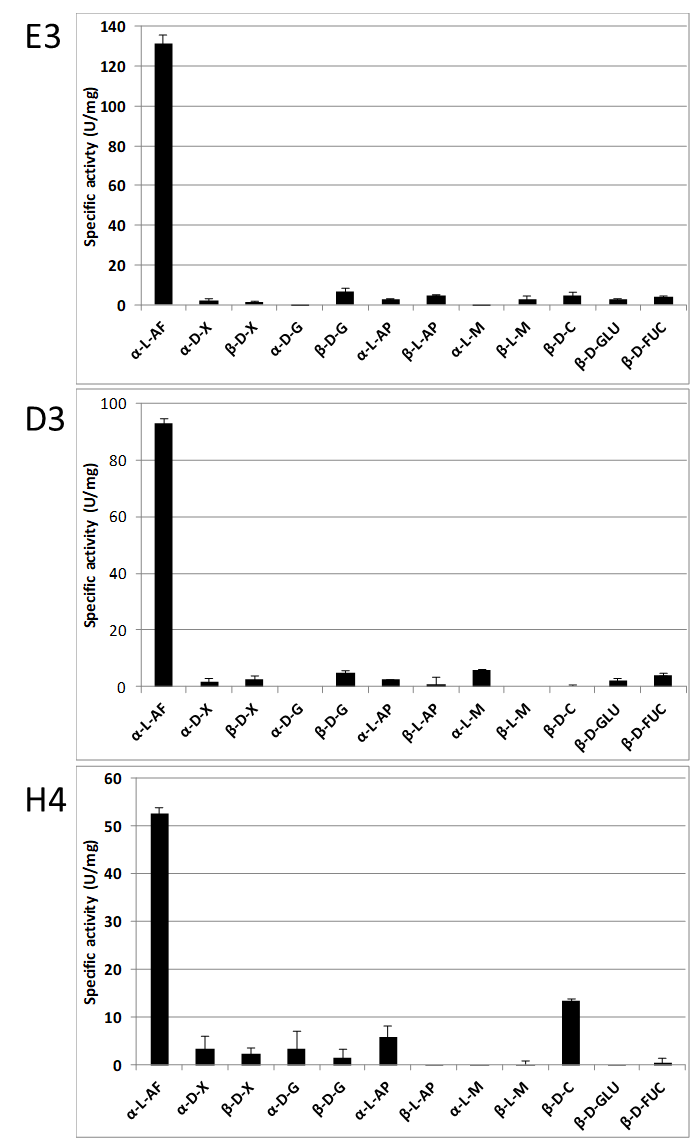

Supplement: Supplementary file 2 — Figure S2. Substrate range of AFases measured on pNP linked glycosides. A) H4, B) E3 and C) D3. Data represents the average of three replicates ± standard error (n = 3). (DOCX 46 kb) [file 12896_2019_510_MOESM2_ESM.docx]
