# Supplementary material for: Characterisation of three novel α-L-arabinofuranosidases from a compost metagenome
Source: BMC Biotechnol. 2019 Apr 18;19:22. doi: 10.1186/s12896-019-0510-1 (PMC6472066; doi:10.1186/s12896-019-0510-1)
Supplement: Supplementary file 2 [file 12896_2019_510_MOESM1_ESM.docx]

D3

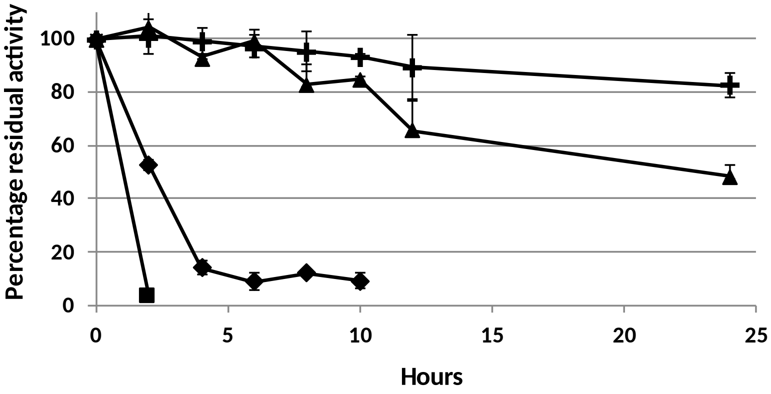
Percentage residual activity
0
20
40
60
80
100
0
5
10
15
20
25
Hours

E3

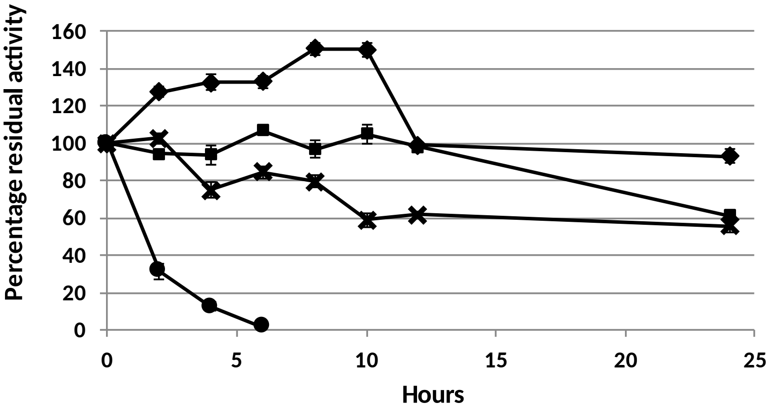
Percentage residual activity
0
20
40
60
80
100
120
140
160
0
5
10
15
20
25
Hours

H4

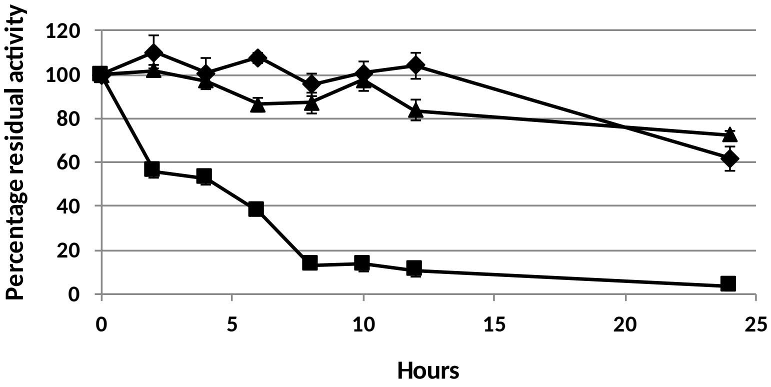
Percentage residual activity
0
20
40
60
80
100
120
0
5
10
15
20
25
Hours
