# Supplementary material for: Characterisation of three novel α-L-arabinofuranosidases from a compost metagenome
Source: BMC Biotechnol. 2019 Apr 18;19:22. doi: 10.1186/s12896-019-0510-1 (PMC6472066; doi:10.1186/s12896-019-0510-1)
Supplement: Supplementary file 3 [file 12896_2019_510_MOESM3_ESM.docx]

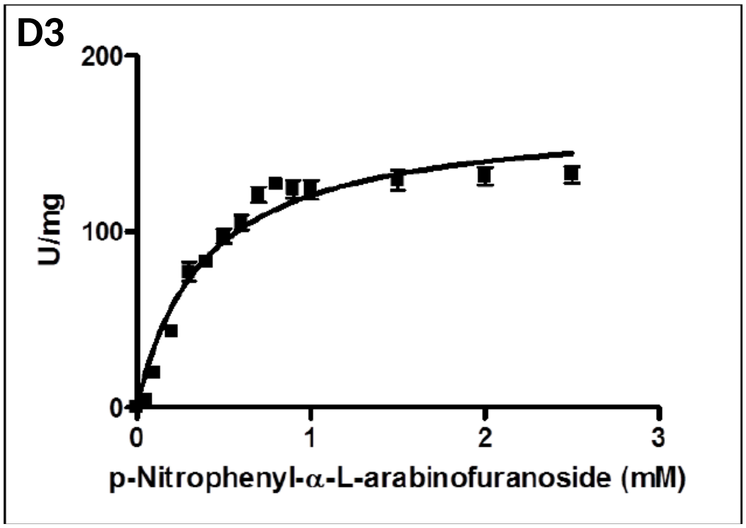

D3
U/mg
200
100
0
0
1
2
3
p-Nitrophenyl-α-L-arabinofuranoside (mM)

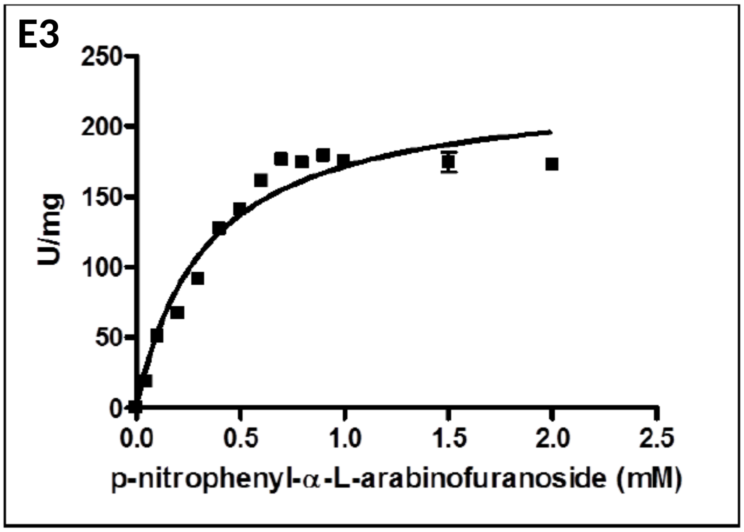

E3
U/mg
250
200
150
100
50
0
0.0
0.5
1.0
1.5
2.0
2.5
p-nitrophenyl-α-L-arabinofuranoside (mM)

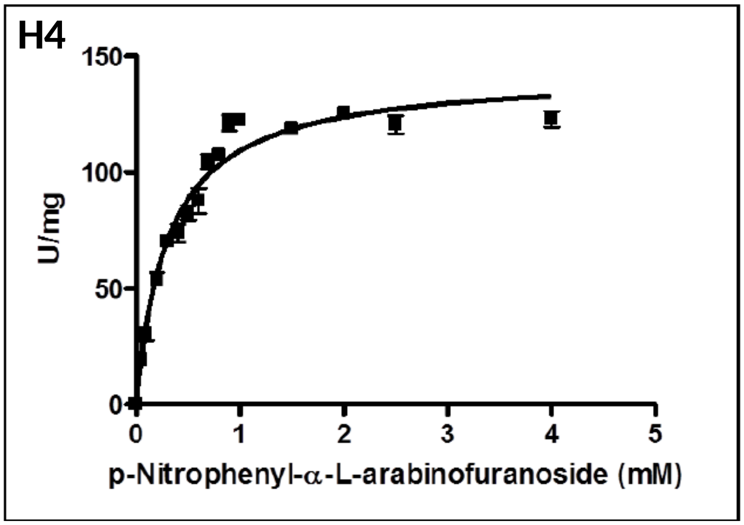

H4
U/mg
150
100
50
0
0
1
2
3
4
5
p-Nitrophenyl-α-L-arabinofuranoside (mM)
